# RETRACTION: Satkara (*Citrus macroptera*) Fruit Protects against Acetaminophen‐Induced Hepatorenal Toxicity in Rats

**DOI:** 10.1155/ecam/9782816

**Published:** 2026-07-27

**Authors:** Evidence-Based Complementary and Alternative Medicine

RETRACTION: S. Paul, M. A. Islam, E. M. Tanvir, et al., “Satkara (*Citrus macroptera*) Fruit Protects against Acetaminophen‐Induced Hepatorenal Toxicity in Rats,” *Evidence-Based Complementary and Alternative Medicine*, 2016, 9470954, https://doi.org/10.1155/2016/9470954.

The above article, published online on 29 February 2016 in Wiley Online Library (https://wileyonlinelibrary.com), has been retracted by John Wiley & Sons Ltd. The retraction has been agreed due to concerns identified with the Figures [1]. Specifically:

• In Figure 6, there are unexpected, repeated areas between panels d and f
• In Figure 6a, there are unexpected, repeated areas when compared to panel c in Figure 6 of [2].
• In Figure 6b, there are unexpected, repeated elements when compared to:
  ◦ Figure 6a of [3].
  ◦ Figure 3b, 3c and 3d of [4].
  ◦ Figure 6b of [2].
  ◦ Figure 7b of [5].
• In Figure 7, panels a, b and g share unexpected, repeated regions. Additionally, these panels also appear similar to panel f of Figure 7 in [2].

Following assessment of the concerns, and in the absence of the original images from the authors in their response, the results and conclusions can no longer be considered reliable. The article is therefore retracted.

Sudip Paul agrees to the retraction, whilst no response was received by the remaining authors.
